# Six-year clinical outcomes of enzyme replacement therapy for perinatal lethal and infantile hypophosphatasia in Korea: Two case reports

**DOI:** 10.1097/MD.0000000000032800

**Published:** 2023-02-10

**Authors:** Insung Kim, Eu-Seon Noh, Min-Sun Kim, Ja-Hyun Jang, Tae Yeon Jeon, Hae Won Choi, Sung Yoon Cho

**Affiliations:** a Department of Public Health Administration, Asan City Health Center, Asan, Korea; b Department of Pediatrics, Samsung Medical Center, Sungkyunkwan University School of Medicine, Seoul, Korea; c Department of Laboratory Medicine and Genetics, Samsung Medical Center, Sungkyunkwan University School of Medicine, Seoul, Korea; d Department of Radiology and Center for Imaging Science, Samsung Medical Center, Sungkyunkwan University School of Medicine, Seoul, Korea; e Department of Orthodontics, The Institute of Oral Health Science, Samsung Medical Center, Sungkyunkwan University School of Medicine, Seoul, Korea.

**Keywords:** alkaline phosphatase, *ALPL*, bone, enzyme replacement therapy, hypophosphatasia

## Abstract

Hypophosphatasia (HPP) is a genetic disease caused by loss-of-function mutations in *ALPL*, which encodes tissue-nonspecific alkaline phosphatase (ALP). Early diagnosis and treatment of perinatal and infantile HPP are important because of their high mortality rates. Enzyme replacement therapy (ERT) using human recombinant tissue-nonspecific ALP asfotase alfa was introduced in Korea in 2016. We report the first experience of ERT over 6 years for perinatal lethal and infantile HPP in Korea.

**Patient concerns:**

The first patient was a 6-week-old Korean boy with a failure to thrive. The second patient was an 8-day-old Korean-Uzbek body with generalized tonic-clonic seizure with cyanosis.

**Diagnoses:**

HPP was suspected in both patients because of the very low level of ALP activity and rachitic findings on radiographs, and the disease was confirmed by Sanger sequencing of the *ALPL* gene.

**Intervention:**

The first patient with infantile HPP started ERT at 21 months of age and the second patient with perinatal HPP started ERT at 30 days of age. Both patients received asfotase alfa (2 mg/kg 3 times per week subcutaneously, adjusted to 3 mg/kg 3 times per week if required) for 6 years.

**Outcomes:**

After 6 years of ERT, radiographic findings and growth standard deviation scores improved in both patients. The second patient showed no evidence of rickets after 3 years of ERT. Mechanical respiratory support and supplemental oxygen were not required after 4.5 years of treatment in the first patient and at 2 months after treatment in the second patient.

**Conclusion:**

Among the 2 patients, the patient who started ERT early had a much better prognosis despite a more severe initial clinical presentation. Our results suggest that early diagnosis and prompt treatment play an important role in improving long-term prognosis and avoiding morbidity and premature mortality in patients with perinatal and infantile HPP.

## 1. Introduction

Hypophosphatasia (HPP) is a genetic disease caused by loss-of-function mutations in *ALPL* located in chromosome 1q36.12. *ALPL* encodes tissue-nonspecific alkaline phosphatase (TNSALP), which degrades inorganic pyrophosphate, an inhibitor of bone and tooth mineralization, to inorganic phosphate. TNSALP also converts pyridoxal 5’-phosphate (PLP) to PL so that the active metabolite of vitamin B6 can pass through the cell membrane.^[1]^ Patients with HPP show clinical features such as skeletal hypomineralization, hypercalcemia, vitamin B6-dependent convulsion, muscular hypotonia, failure to thrive, and developmental delay. HPP is classified into 6 forms according to the onset and severity of manifestations: perinatal lethal, perinatal benign, infantile, childhood, adult, and odontohypophosphatasia. The prevalence of perinatal lethal and infantile HPP is 1 in 100,000 to 900,000.^[2,3]^ Early diagnosis and appropriate medical intervention for perinatal and infantile HPP are important because the majority of patients with perinatal lethal HPP die during the perinatal period, and infantile HPP has a mortality rate of approximately 50% during infancy.^[2]^ Since enzyme replacement therapy (ERT) with human recombinant TNSALP asfotase alfa showed clinical and radiographic improvements in patients with life-threatening perinatal lethal or infantile HPP in 2012,^[4]^ this drug has become available in many countries and was introduced in Korea in 2016. We aimed to describe the 6-year outcomes of 2 children with infantile and perinatal lethal HPP after ERT.

## 2. Case reports

### 2.1. Patient 1

Patient 1, a 6-week-old Korean boy, visited Samsung Medical Center in 2014 because of failure to thrive and poor feeding. He was born at 40 weeks and 5 days of gestation via vaginal delivery without perinatal issues. Although his birth weight was 3.75 kg, length 51 cm, and head circumference 35 cm, at the first outpatient visit at our center at 6 weeks of age, he showed catch-down growth of 3.7 kg in weight, 56.2 cm in length, and 36 cm in head circumference. He was hospitalized for 3 weeks and was treated with hydration and a low-calcium formula owing to hypercalcemia. Due to persistently low serum ALP levels with rachitic findings on radiographs, HPP was suspected. At 12 months of age, tracheostomy, gastrostomy, and fundoplication were performed because of life-threatening aspiration pneumonia. Home ventilation was continued after discharge. The patient’s clinical course up to the ERT baseline has been previously described in detail (Table 1).^[5]^

**Table 1:** Characteristics of 2 children with hypophosphatasia at starting enzyme replacement therapy.

Characteristic	Patient 1	Patient 2
Variants in *ALPL*	c.334G > A (p.Gly112Ser)	c.1025A > G (p.Glu351Gly)
	c.1039C > T (p.Gln347*)	c.1599delT (p.Leu520Argfs*86)
Sex	Male	Male
Age		
At 1st visit	6 wk	8 d
At diagnosis	10 mo	21 d
At start of ERT	21 mo	30 d
ERT baseline		
Laboratory results		
Serum ALP (U/L)	5 (RR, 150–369)	5 (RR, 134–518)
Plasma PLP (nmol/L)	6112 (RR, 15–73)	4207 (RR, 15–73)
Serum total Ca (mg/dL)	10.8 (RR, 9.2–10.5)	10.5 (RR, 8.5–11.0)
Serum P (mg/dL)	5.6 (RR, 4.3–6.8)	6.7 (RR, 4.8–8.4)
Serum PTH (pg/mL)	1.6 (RR, 11–59)	7.8 (RR, 11–59)
Clinical status		
Respiration	Tracheostomy and home ventilator	Endotracheal intubation and mechanical ventilator
Feeding	Gastrostomy	Feeding tube
Gross motor function	Age-equivalent by <1 mo	Not applicable; neonate

ALP = alkaline phosphatase, Ca = calcium, ERT = enzyme replacement therapy, P = phosphorus, PLP = pyridoxal 5’-phosphate, PTH = parathyroid hormone, RR = reference range.

He was diagnosed with HPP at 10 months of age, with c.334G > A (p.Gly112Ser) inherited from his Korean mother and c.1039C > T (p.Gln347*) inherited from his Korean father based on Sanger sequencing of the *ALPL* gene. Variants were identified by comparing the patient sequence with the reference sequence for the *ALPL* gene (GenBank accession number, NM_000478.4). After the diagnosis of HPP, he started ERT at 21 months of age via a compassionate route. Asfotase alfa (Strensiq®; Alexion Pharmaceuticals, CN) was used for ERT at an initial dose of 2 mg/kg via subcutaneous injection 3 times weekly. If there was no improvement or worsening of clinical indicators such as respiratory status, growth, or radiographic findings, the dose of ERT was increased to 3 mg/kg 3 times weekly. When the effect was confirmed, the absolute drug dose was maintained such that the dose per body weight gradually decreased as the weight increased. The drug dose was not reduced to <2 mg/kg 3 times weekly during ERT. Results of skeletal simple radiograph with quantitative measurements (Rickets Severity Scale [RSS]^[6]^ and Radiographic Global Impression of Change [RGI-C]^[7]^), laboratory tests (ALP, PLP, calcium, phosphorus, and parathyroid hormone [PTH] levels), growth (weight, height, head circumference), development (Korean Developmental Screening Test for infants and children from 4 to 71 months, Bleck score, 6-minute walking test), respiration, feeding, and dentition were monitored at baseline and at intervals of <6 months before 1 year of treatment and for approximately every 6 months after 1 year of treatment. Complications of HPP including seizure, craniosynostosis, and nephrocalcinosis were monitored during ERT.

**Figure 1.**
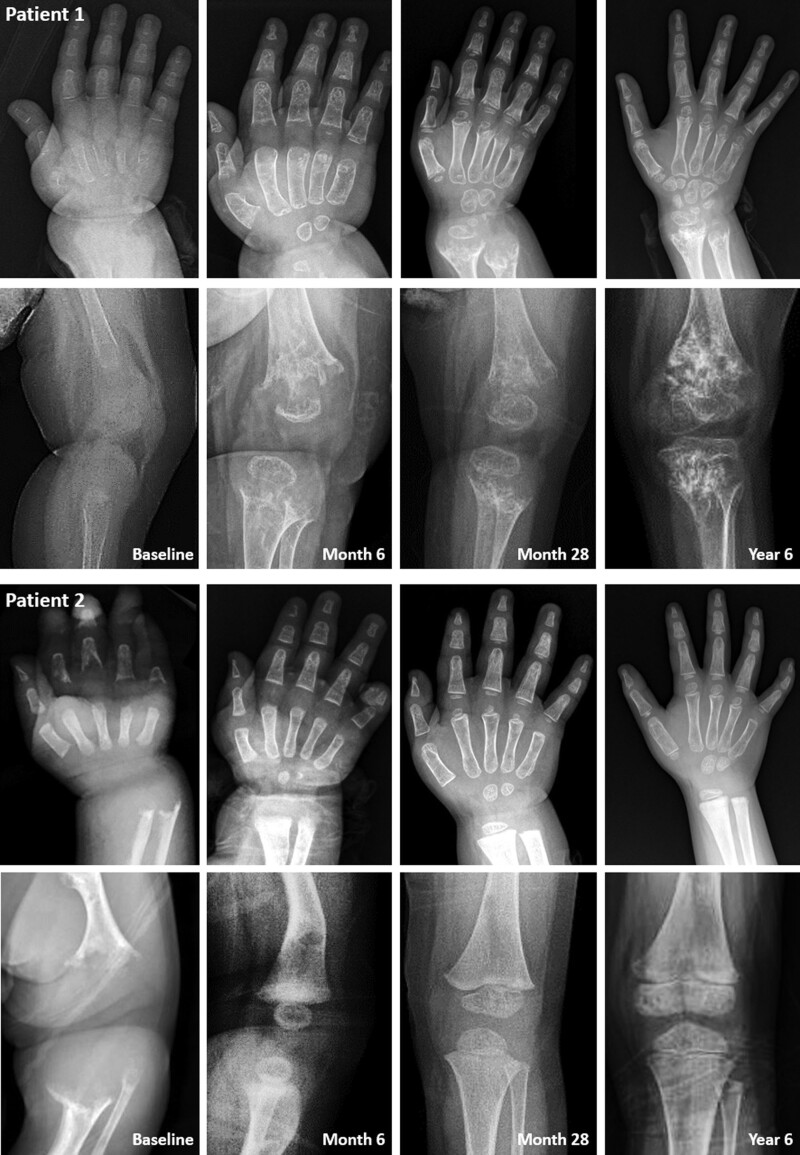
Serial wrist and knee plain radiographs of Patient 1 and 2. The improvement in radiographic findings was more pronounced in Patient 2 compared to Patient 1.

**Figure 2.**
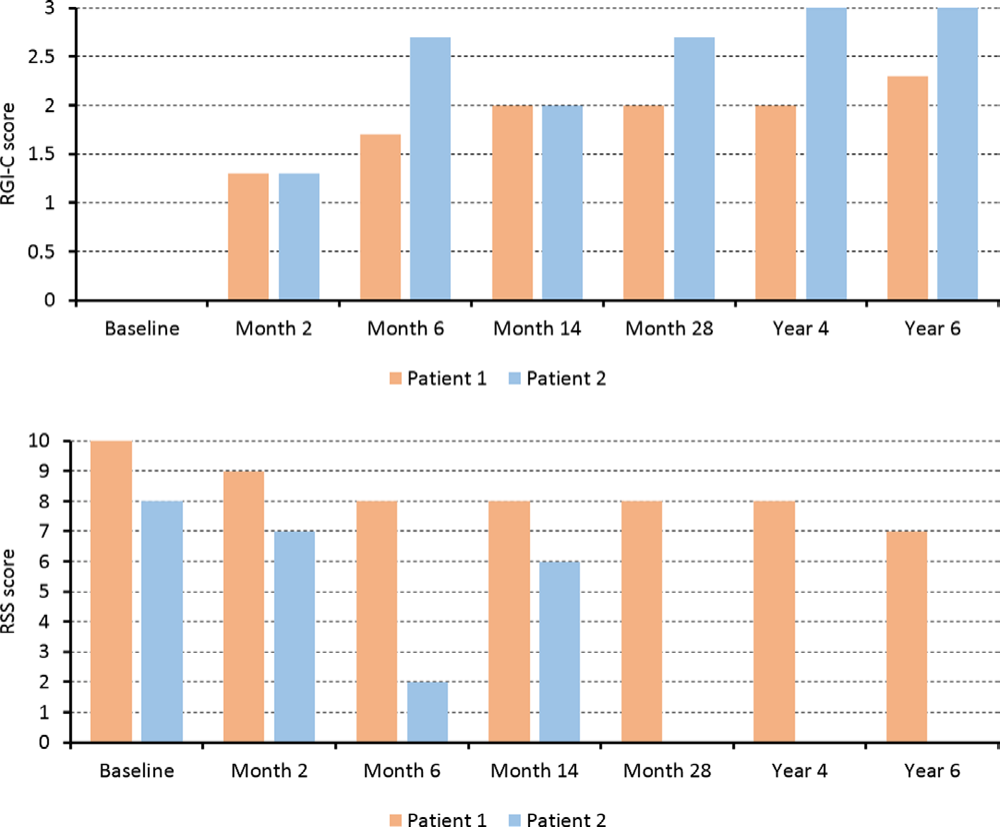
Radiographic scores during enzyme replacement therapy. RSS = Rickets Severity Scale, RGI-C = Radiographic Global Impression of Change.

**Figure 3.**
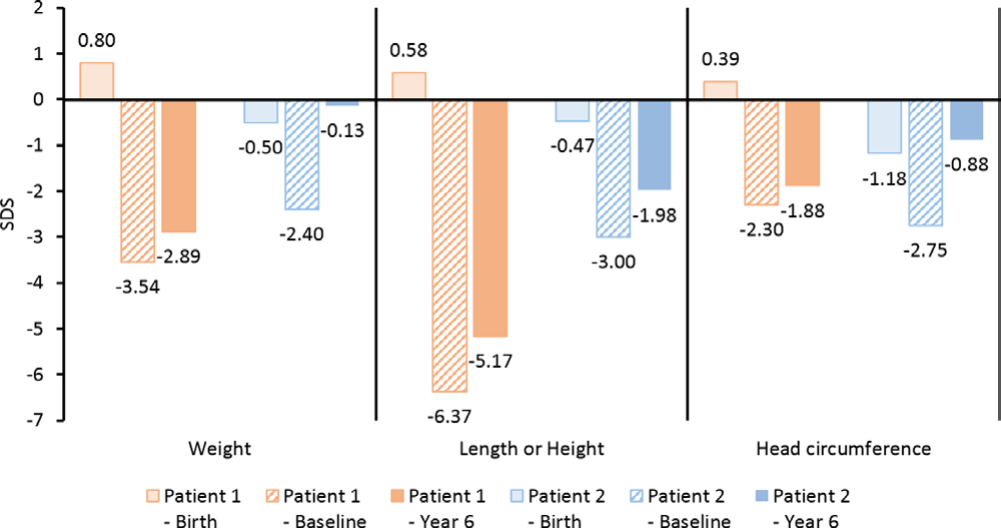
Changes of body measurements after 6 years of enzyme replacement therapy. SDS = standard deviation score.

At baseline, the weight, length, and head circumference of Patient 1 showed a <−2 standard deviation score (SDS). Serum total calcium and serum phosphorus levels were around the normal limits, and he had low serum ALP activity (5 U/L), suppressed serum PTH, and elevated PLP. After starting ERT, serum calcium, phosphorus, and PTH levels remained within the normal ranges, ALP activity was greatly increased, and PLP was remarkably decreased (time series graphs of laboratory findings are provided in Supplementary Figure S1, Supplemental Digital Content, http://links.lww.com/MD/I409). Serial plain images showed improvement in irregular osteolytic bone change with change in metaphyseal fraying, physeal widening, and bowing of extremities as well as improved general mineralization. The RSS and RGI-C scores improved with a gentle slope (Figs. 1 and 2). Respiratory support was not required 54 months after treatment. After 6 years of ERT, Patient 1 showed RGI-C +2.3, height *Z* score +1.2, and weight *Z* score +0.65 (Figs. 2 and 3) (more detailed serial radiographic images are provided in Supplementary Figure S2, Supplemental Digital Content, http://links.lww.com/MD/I410). Although the weight and height were <−2 SDS, the growth curve was parallel to a normal curve (detailed growth curves are provided in Supplementary Figure S3, Supplemental Digital Content, http://links.lww.com/MD/I411). At the most recent outpatient visit, Patient 1 was able to balance on his knees and was trying oral feeding while monitoring regurgitation. Intraoral examination revealed that 13 of the 20 primary teeth exfoliated prematurely during ERT (Table 2).

**Table 2:** Summary of clinical status over 6 years of enzyme replacement therapy.

Clinical status over 6 yr of ERT		
Age at 6 yr of ERT	7 yr 8 mo	6 yr 1 mo
Respiration		
Status	Ambient air	Ambient air
Respiratory support end*	Month 54 of treatment	Day 57 of treatment
Feeding	Gastrostomy	Oral feeding
Gross motor development		
K-DST	0 (The 71-mo test was used)	15 (−2 SDS cut off point 16) (the 71-month test was used)
Bleck score	0	9
6-minute walking test	Not able to perform	327.2 m
Dental status (number of primary teeth with premature exfoliation)	13	10
	8 incisors	7 incisors
	2 canines	2 canines
	3 molars	1 molar
Complications and adverse events during ERT		
Respiratory disorder†	2 pneumonia events 1 severe desaturation event	No
Seizure	No	No seizure after 1st episode
Cranoisynostosis	No	No
Nephrocalcinosis	1 ureter stone event Calyceal stones on US	Mild medullary nephrocalcinosis on US
Adverse events	1 local redness and swelling at the injection site	No

ERT = enzyme replacement therapy, K-DST = Korean Developmental Screening Test, SDS = standard deviation score, US = ultrasonography.

* Respiratory support includes mechanical support during hospitalization, home ventilator use, and simple oxygen supply. † In Patient 1, no hospitalization due to respiratory disorder exceeded 5 days. There was no respiratory complication requiring hospitalization after week 22 of treatment.

### 2.2. Patient 2

Patient 2, an 8-day-old Korean-Uzbek boy, was hospitalized in the neonatal intensive care unit in 2016 because of generalized tonic-clonic seizure with cyanosis. He was delivered at 41 weeks of gestation with a birth weight of 3.1 kg, length of 49 cm, and head circumference of 33 cm. Endotracheal intubation was performed, and mechanical ventilation was initiated for respiratory distress. A skeletal survey showed generalized osteopenia, thin ribs, and a tongue-like protrusion of radiolucency on the metaphyses of the long bones. He was transferred to the Samsung Medical Center at 20 days of age for further evaluation and management. His serum total calcium level was within the normal range; however, his ALP activity was very low. The clinical course of this patient before transfer to our center has been previously described (Table 1).^[8]^

Patient 2 was diagnosed with HPP at 21 days of age, with c.1052A > G (p.Glu351Gly) inherited from his Uzbek mother and c.1559delT (p.Leu520Argfs*86) inherited from his Korean father. ERT was initiated immediately after diagnosis at the age of 30 days. The diagnostic method, treatment protocol, and monitoring during ERT were the same as those used for Patient 1.

Little growth occurred before starting ERT, and all growth curves were under the reference of −2 SDS. Similar to Patient 1, low serum ALP activity (5 U/L), suppressed serum PTH, and elevated PLP were observed in the laboratory test at baseline, and the results remained within the normal range with increased levels of ALP activity after starting ERT (time series graphs of laboratory findings are provided in Supplementary Figure S1, Supplemental Digital Content, http://links.lww.com/MD/I409). Serial radiographic images and indices improved more dramatically than in Patient 1 (Figs. 1 and 2) (more detailed serial radiographic images are provided in Supplementary Figure S2, Supplemental Digital Content, http://links.lww.com/MD/I410). There was no clinical or radiographic evidence of rickets after 3 years of ERT. Respiratory support was no longer needed after extubation on day 57 of treatment. This patient showed RGI-C +3, height *Z* score +1.02, and weight *Z* score +2.27 at 6 years of treatment (Figs. 2 and 3). The growth curves were near the mean of the normal growth curves within −2 SDS (detailed growth curves are provided in Supplementary Figure S3, Supplemental Digital Content, http://links.lww.com/MD/I411). At the most recent outpatient visit, Patient 2 was able to run and play. Intraoral examination revealed that 10 of the 20 primary teeth exfoliated prematurely during ERT (Table 2).

## 3. Discussion

We described the clinical outcomes of 2 children with infantile and perinatal lethal HPP who underwent ERT over a duration of 6 years. Although clinical improvements were noted, there were differences in clinical features and treatment courses between the 2 patients. Age at ERT onset and genotype have an important influence on clinical severity and treatment outcome.

The most important difference between Patients 1 and 2 was the age at ERT onset. Because Patient 1 was the first patient to receive ERT with asfotase alfa in Korea, it took several months to establish a compassionate route. Therefore, Patient 1 started ERT at the age of 21 months. Based on this experience, Patient 2 started ERT at the age of 30 days, resulting in a much better overall clinical status than that of Patient 1, despite a more severe initial clinical presentation. Clinical guidelines emphasize the early initiation of ERT before severe deterioration of respiratory function and bone mineralization for better prognosis in patients with perinatal or infantile HPP.^[9]^ According to reviews of the results of multicenter, multinational, phase II interventional studies of asfotase alfa treatment for perinatal and infantile HPP (median duration of treatment, 2.7 years), 37 treated patients were compared with 48 patients who did not undergo such treatment.^[10]^ The treated patients showed significantly improved survival rates (95% vs 42% at age 1 year and 84% vs 27% at age 5 years), especially those who needed ventilator support (76% vs 5%).

Previous 7-year long-term outcomes of asfotase alfa treatment in 11 perinatal or infantile HPP patients showed improved bone mineralization indices (median RSS score 0.5 and median RGI-C score +2.0 at year 6 of treatment) and *Z* scores of length or height and weight (mean *Z* score change from baseline; length +1.37 and weight +2.02 at year 6 of treatment). None of the patients who completed the study required respiratory support after completion of treatment. The patient with the longest duration of mechanical ventilation was started on ERT at the age of 33 months. She was weaned off mechanical ventilation after 3.5 years of treatment and underwent tracheocutaneous fistula closure after 5.5 years of treatment.^[11]^ Our patients had similar outcomes. Although case 1 showed relatively slow recovery, we expect gradual improvement similar to the patients in other studies.^[10,11]^

HPP has been associated with 386 mutation variants in ALPL (HGMD® Professional 2021.4). Most of these mutations are missense variants, though there are also nonsense, frameshift, and inframe deletion mutations. Unlike other mutations that cause severe enzyme deactivation, missense variants show varying degrees of residual activity of TNSALP, which is related to the clinical heterogeneity of HPP.^[12]^ In our report, *ALPL* mutation c.1039C > T (p.Gln347*) in Patient 1 and c.1052A > G (p.Glu351Gly) in Patient 2 had not been reported previously. One Belgian neonate with the same c.334G > A (p.Gly112Ser) variant, as found in Patient 1, was reported to have a more severe perinatal form of HPP in 2004.^[13]^ The mutation found in Patient 2 and his Korean father, c.1559delT (p.Leu520Argfs*86), is now well known as a common mutation in Japanese patients with HPP, with approximately 1/480 carriers in the Japanese population.^[3]^ Many HPP patients harboring this mutation have shown phenotypes consistent with the perinatal lethal form,^[14,15]^ and in vitro assays revealed significantly decreased activity of TNSALP.^[16]^

Patients with HPP have functional problems with eating because their teeth are weak and exfoliated early. In one study, perinatal lethal and infantile HPP patients who started ERT during infancy showed less premature exfoliation of the teeth than those who started ERT after infancy.^[17]^ In our patients, the number of lost primary teeth was fewer in Patient 2, who started ERT at an earlier age, which is compatible with the results of a previous study. However, we believe that ERT cannot prevent all dental problems. Further research on the effects of ERT on primary and permanent dentition is required.

## 4. Conclusion

As in other studies, we confirmed improvements in the respiratory status, bone mineralization, and growth after starting ERT. In addition, despite the severity of initial clinical presentation, the patient who started ERT early had a much better prognosis. To the best of our knowledge, our 2 patients are the only patients who have been treated with ERT in Korea, and a considerable number of patients with HPP likely remain undiagnosed considering the size of the Korean population. Therefore, it is important to suspect HPP early if a decreased ALP level is accompanied by abnormal plain radiographic findings, respiratory distress, and failure to thrive for early diagnosis and treatment. Additional ERT data for HPP patients are needed to manage ERT issues such as adulthood disease, high treatment costs, and dental problems.

## Acknowledgments

We thank our patients and their families and all the clinical and research laboratory staff. This study was supported by Samsung Medical Center Grants #2021R1G1A1092117 and #SMO1220351.

## Author contributions

**Conceptualization:** Insung Kim, Sung Yoon Cho.

**Investigation:** Eu-Seon Noh, Min-Sun Kim, Tae Yeon Jeon, Hae Won Choi, Sung Yoon Cho.

**Methodology:** Eu-Seon Noh, Min-Sun Kim, Sung Yoon Cho.

**Supervision:** Sung Yoon Cho.

**Visualization:** Insung Kim.

**Writing – original draft:** Insung Kim.

**Writing – review & editing:** Insung Kim, Ja-Hyun Jang, Tae Yeon Jeon, Hae Won Choi, Sung Yoon Cho.

## Supplementary Material






