# Supplementary material for: Six-year clinical outcomes of enzyme replacement therapy for perinatal lethal and infantile hypophosphatasia in Korea: Two case reports
Source: Medicine (Baltimore). 2023 Feb 10;102(6):e32800. doi: 10.1097/MD.0000000000032800 (PMC9907957; doi:10.1097/MD.0000000000032800)

**Supplementary Figure 1** Changes of laboratory results of Patient 1 and Patient 2. In each graph, the black solid vertical line indicates date of birth, the black dashed vertical line indicates onset of enzyme replacement therapy, and colored area indicates the reference range. * Blue lines indicate serum total calcium level and green lines indicate serum phosphorus level. Ca = serum total calcium, P = serum phosphorus, PLP = pyridoxal 5'-phosphate, PTH = parathyroid hormone.

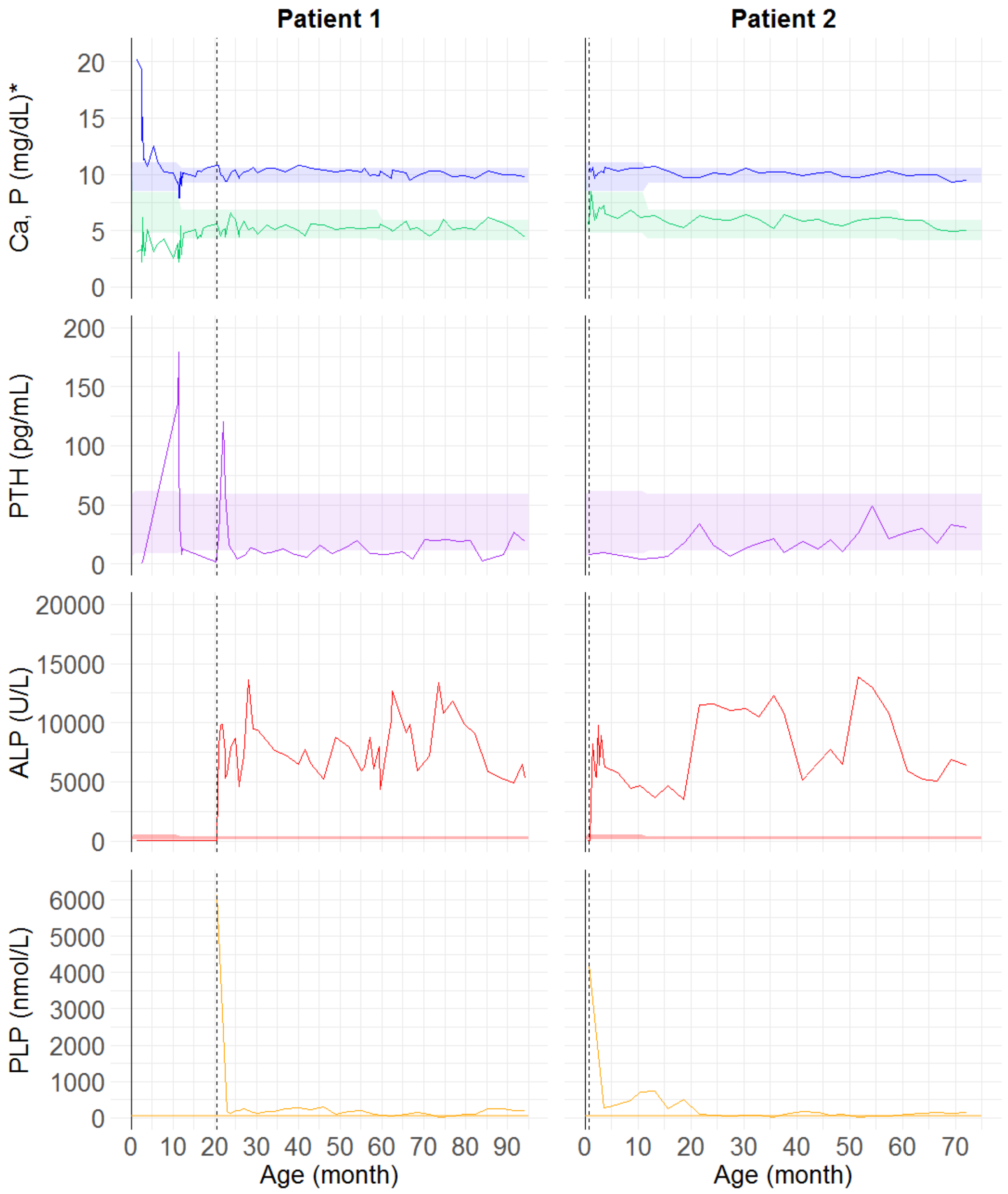

Supplement: Supplementary file 1 [file medi-102-e32800-s001.pdf]
