# Supplementary material for: Six-year clinical outcomes of enzyme replacement therapy for perinatal lethal and infantile hypophosphatasia in Korea: Two case reports
Source: Medicine (Baltimore). 2023 Feb 10;102(6):e32800. doi: 10.1097/MD.0000000000032800 (PMC9907957; doi:10.1097/MD.0000000000032800)

Supplementary Figure 2A Serial chest simple radiographs of Patient 1 and 2. Thin ribs were showed in baseline radiographs of both patients. It can be seen that the thickness of the ribs increased significantly even after two months of enzyme replacement therapy.

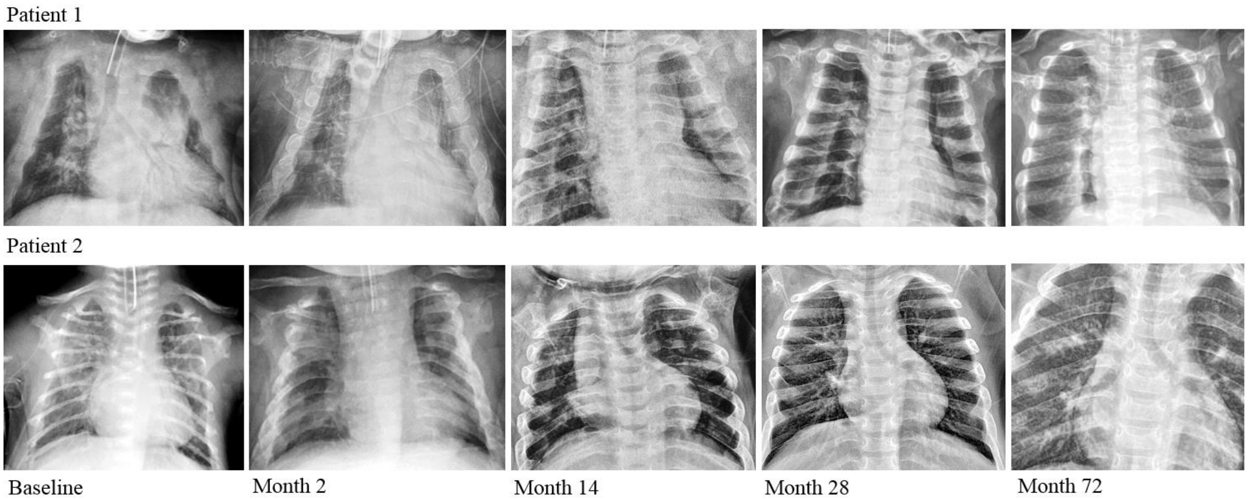

Supplementary Figure 2B Serial skull simple radiographs of Patient 1 and 2. Both patients showed severe anterior fontanelle widening at baseline. Note that Patient 1 was 21 months old at baseline. This findings improved after enzyme replacement therapy. However, while Patient 1 showed irregular margins in the shape and surface of the skull, Patient 2 went through a natural fontanelle closing.

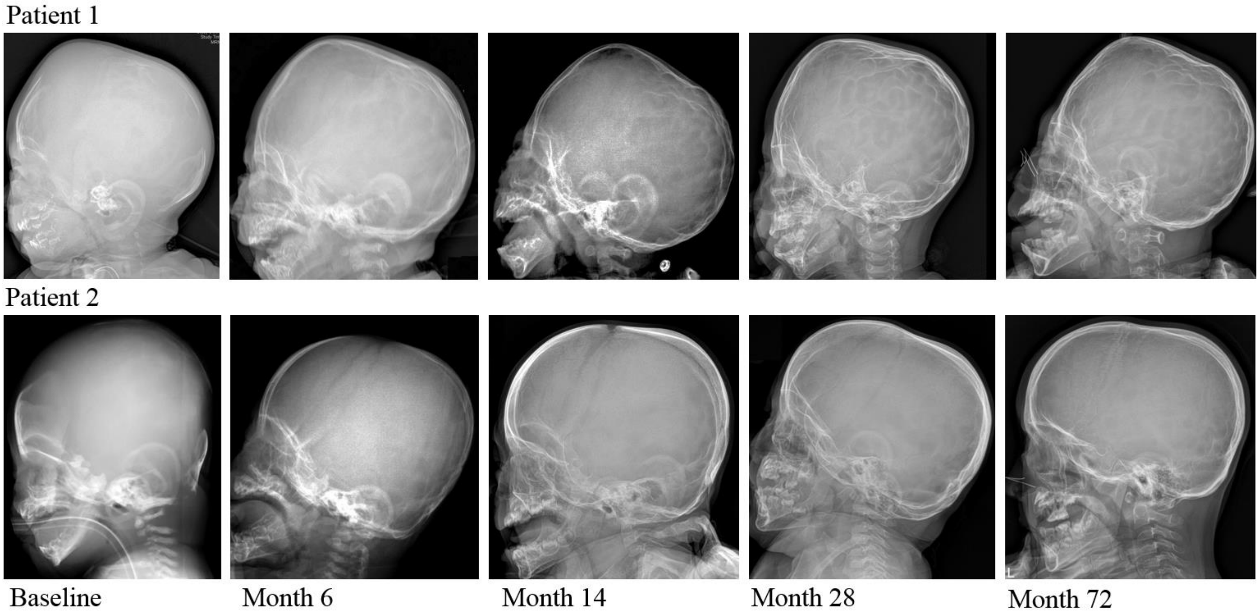

Supplementary Figure 2C Serial spine simple radiographs of Patient 1 and 2. Anterior beaking of vertebral bodies were observed at baseline. Bone density and height of vertebral bodies increased during enzyme replacement therapy.

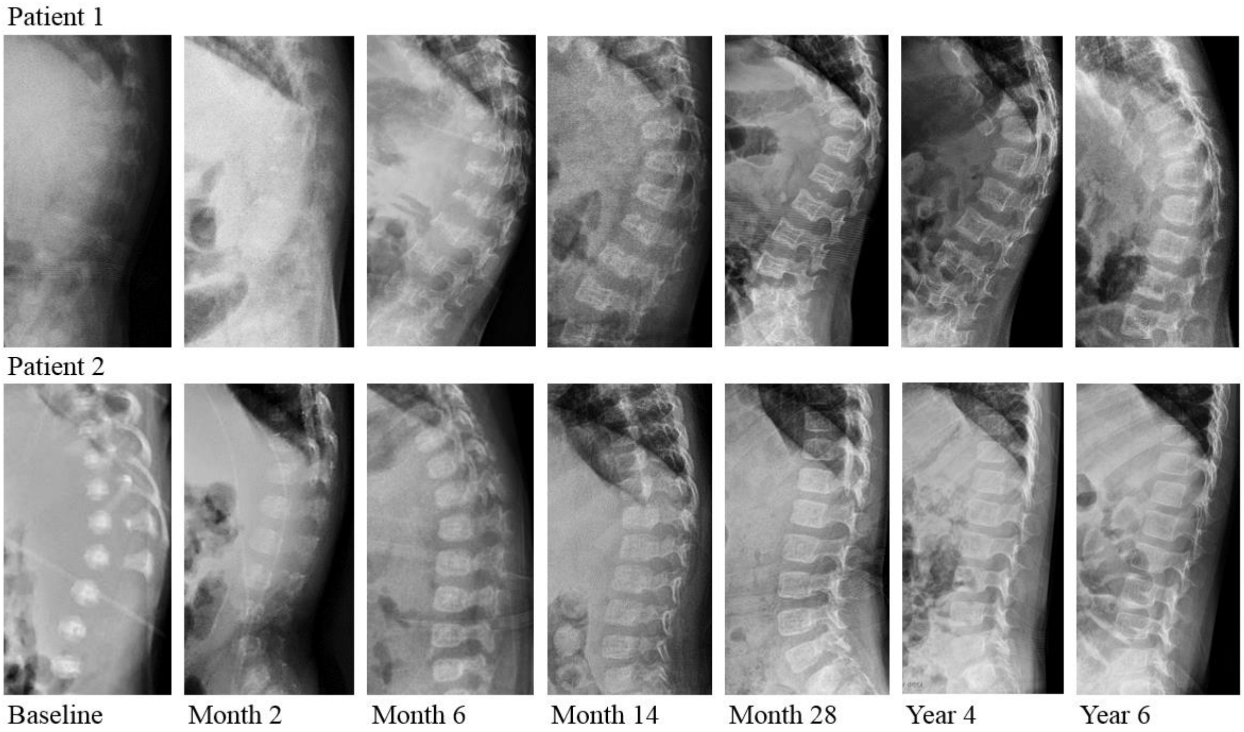

Supplementary Figure 2D Serial leg simple radiographs of Patient 1 and 2. Images showed gradual improvement of mineralization of the entire bones with improved metaphyseal irregular fraying, physeal widening, and bowing of extremities during enzyme replacement therapy. Patient 1 had a slower recovery compared to Patient 2 and still showed irregularity of provisional zone of calcification in the knee after six years of treatment.

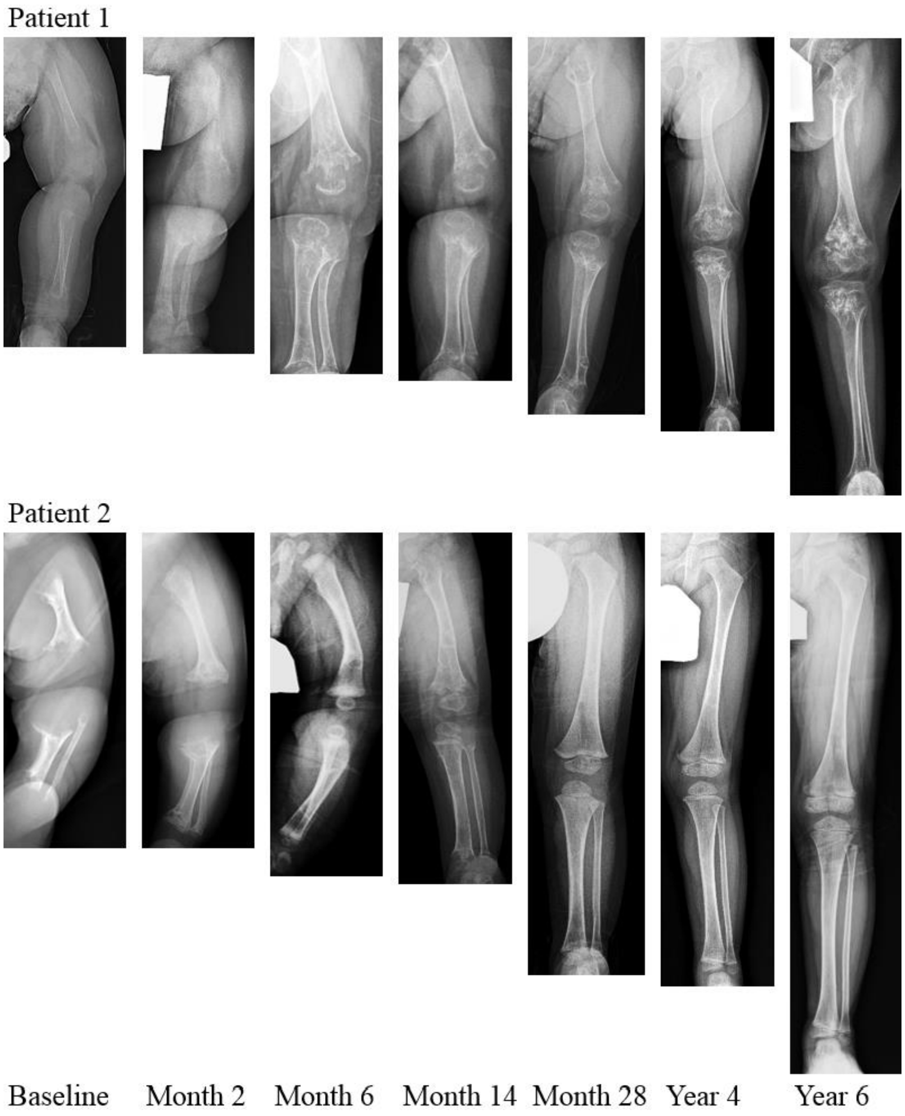

Supplementary Figure 2E Serial hand and wrist simple radiographs of Patient 1 and 2. Irregular osteolytic bone change with metaphyseal fraying in phalanges, metacarpal bones, radius and ulnar improved after enzyme replacement therapy with progression of bony mineralization.

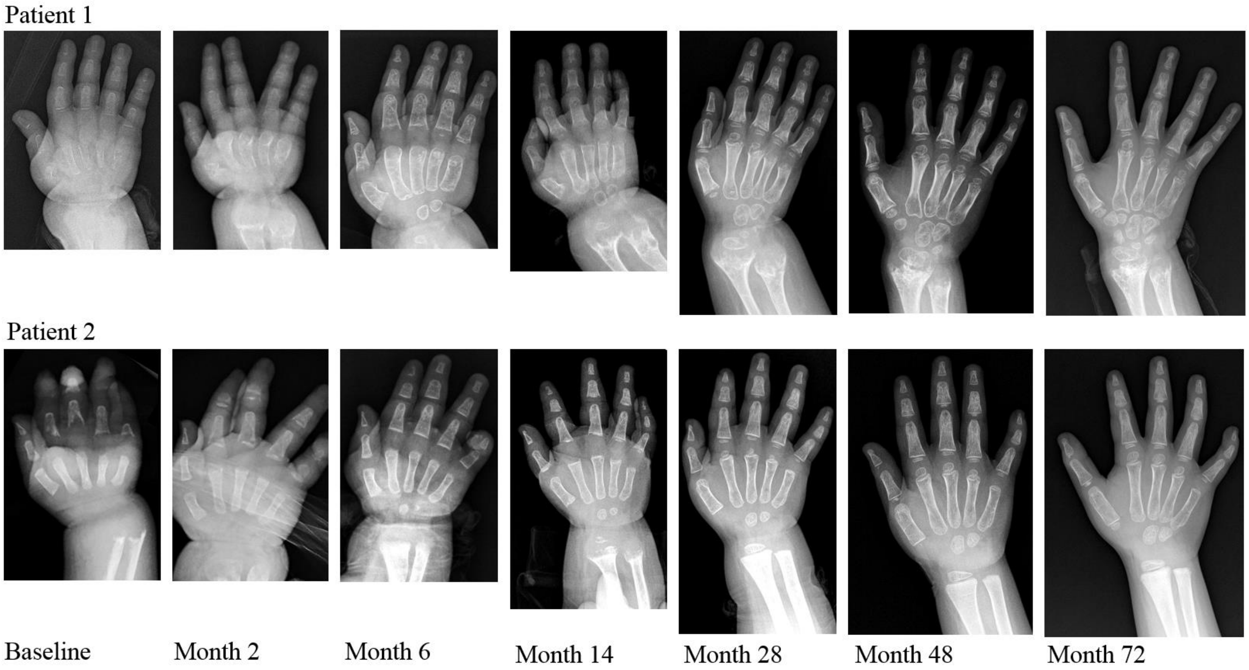

Supplement: Supplementary file 2 [file medi-102-e32800-s002.pdf]
