# Supplementary material for: Six-year clinical outcomes of enzyme replacement therapy for perinatal lethal and infantile hypophosphatasia in Korea: Two case reports
Source: Medicine (Baltimore). 2023 Feb 10;102(6):e32800. doi: 10.1097/MD.0000000000032800 (PMC9907957; doi:10.1097/MD.0000000000032800)

Supplementary Figure 3 Growth curves of Patient 1 and Patient 2. In each growth curve, the black solid vertical line indicates date of birth and the black dashed vertical line indicates start of enzyme replacement therapy. The black dot graph indicates data of patients, and colored line graphs indicate reference data: a central bold solid line indicates mean, dashed lines indicate ±1 standard deviation, outer solid lines indicate ±2 standard deviation, and dotted lines indicate ±3 standard deviation.

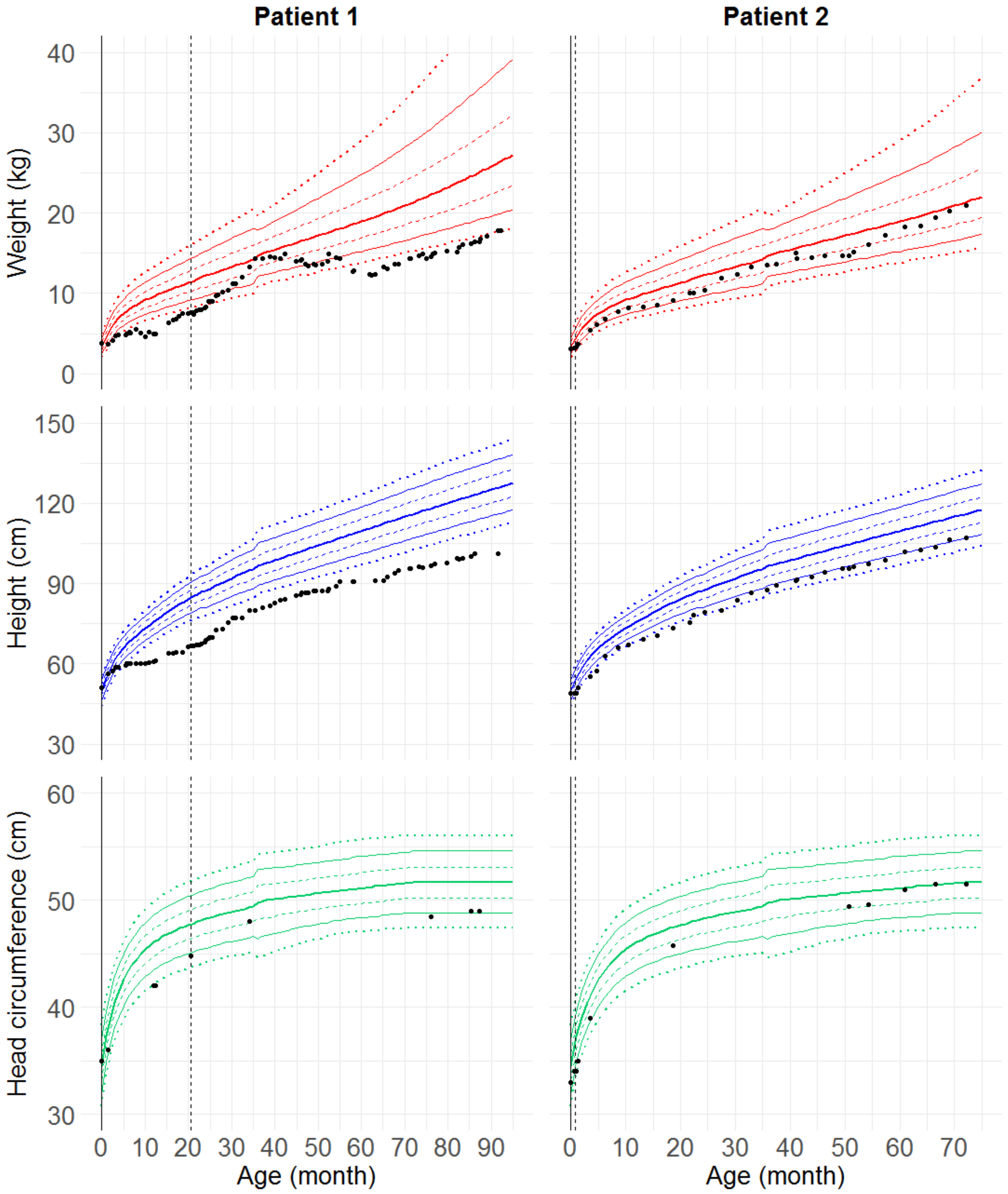

Supplement: Supplementary file 3 [file medi-102-e32800-s003.pdf]
